# Surface-Plasmon-Resonance Amplification of FMD Detection through Dendrimer Conjugation

**DOI:** 10.3390/s24020579

**Published:** 2024-01-17

**Authors:** Seung Jun Jung, Jin-Won Park

**Affiliations:** Department of Chemical and Biomolecular Engineering, College of Energy and Biotechnology, Seoul National University of Science and Technology, Seoul 01811, Republic of Korea; tbwn621@naver.com

**Keywords:** foot-and-mouth disease, dendrimer, aptamer, surface plasmon resonance, sensitivity

## Abstract

The amplification of the surface plasmon resonance (SPR) sensitivity for the foot-and-mouth disease (FMD) detection was studied using Poly(amidoamine) (PAMAM) succinamic-acid dendrimers. The dendrimers were conjugated with the complementary annealed with the aptamers capable of binding specifically to FMD peptides. The tethered layer of the dendrimer-conjugated double-stranded(ds)-aptamers was formed on the SPR sensor Au surface via a thiol bond between the aptamers and Au. After the tethered layer was formed, the surface was taken out of the SPR equipment. Then, the ds-aptamers on the surface were denatured to collect the dendrimer-conjugated single-stranded(ss)-complementary. The surface with only the remaining ss-aptamers was transferred again to the equipment. Two types of the injections, the FMD peptide only and the dendrimer-conjugated ss-complementary followed by the FMD peptides, were performed on the surface. The sensitivity was increased 20 times with the conjugation of the dendrimers, but the binding rate of the peptides became more than two times slower.

## 1. Introduction

FMD is one of the world’s most economically important viral diseases affecting cloven hoofed animals [1]. The infection leads vesicular lesions in and around the mouth and on the feet, causing the reluctance of an animal to eat or move [2]. Since FMD is highly transmissible, an accidental introduction of the FMD virus in a susceptible population can result in an abrupt outbreak of the disease [3]. FMD sensing methods were categorized into nucleic-acid detection and serological methods [4]. Nucleic-acid detection is a complementary-binding-based technique-commonly reverse transcription (RT)-polymerase chain reaction, RT-loop-mediated isothermal amplification, and RT-recombinase polymerase amplification, whereas serological methods are immune-binding-based techniques, i.e., enzyme-linked immunosorbent assay (ELISA), liquid-phase blocking ELISA, and sulfated polysaccharide-coating ELISA. Both methods have become accurate, fast, and sensitive on a pico-molar scale [4].

SPR is an analytical tool for real-time detection in which an optical signal is proportional to the concentration of an analyte capable of specific binding [5,6]. Therefore, SPR has been used to sense biomolecules such as nucleic acids and proteins on the nanomolar scale [7,8]. Aptamers are small sequences of DNA or RNA that fold into well-defined and stable structures to interact specifically with molecular targets [9]. The use of SPR with aptamers consists of two types—direct (one-site binding) mode and sandwich (two-site binding) mode [8]. The sensitivity was improved with the addition of the binding, according to the transition from the former to the latter mode. The addition was acquired mainly through nanostructures including magnetic particles, gold nanomaterials, quantum dots, and graphene [7,10,11,12,13,14].

The PAMAM dendrimer is a sphere-like polymer made of repetitively branched subunits of amide and amine functionality [15]. The dendrimer immobilized on a surface can prevent non-specific adsorption to the surface due to its hydrophilicity caused by the subunits [16]. Furthermore, functional groups such as hydroxyl, carboxylic acid, or amine can be functionalized on the dendrimer for subsequent conjugations with the biomolecules of interest [17,18,19,20,21,22]. Therefore, dendrimers have been widely used on various substrates, including oxide, polymer, lipid, and gold to develop biosensing devices [18,23,24,25,26,27,28,29]. In this study, we aim to enhance FMD detection sensitivity of a conventional aptamer-based SPR through the conjugation between PAMAM dendrimers and aptamers.

## 2. Materials and Methods

The PAMAM succinamic-acid dendrimer (4th generation, molecular weight 20,646.87) was purchased from Sigma Aldrich (St. Louis, MO, USA). The aptamers annealed with the complementary strand were custom-synthesized by IDT (Coralville, IA, USA). The aptamer sequence was 5′-dithiol-C3spacer-TGA-ATA-TCT-CTT-CTA-CCT-CCT-CTC-CTC-CCT-TTA-CTT, and the complementary strands were amine-functionalized at the 5′-end. FMD peptides were from Genscript (Piscataway, NJ, USA) [30]. A 0.2 mL solution of 48 mM 1-ethyl-3-(3-dimethylaminopropyl)-carbodiimide and 12 mM N-hydroxysuccinimide was added to a 0.1 mL dendrimer solution (10 wt% in water), followed by 15-min incubation to activate the carboxylic-acid of the dendrimers. The activated dendrimers were mixed with a double volume of 100 nM double-stranded aptamers (ds-aptamers) in 10 mM MES buffer for the conjugation between the dendrimers and the complementary of the aptamers. Dialysis was performed to remove unreacted materials using a 1 kDa MWCO membrane from Thermo Fisher Scientific (Waltham, MA, USA). The diameters of the dendrimers before and after the conjugation respectively were measured using dynamic light-scattering (Otsuka Electronics Co., Ltd., Osaka, Japan).

SPR measurements were performed according to the procedures described in a previous publication [13]. A bare SPR sensor Au surface was acquired from BIAcore SA (Little Chalfont, Buckinghamshire, UK). Immediately prior to use, the Au surface was cleaned in a 4:1 solution of 96% sulfuric acid and 30% hydrogen peroxide at 60–80 °C for 5 min. The Au surfaces was dried in nitrogen and transferred to a SPR equipment (BIAcore 3000, Little Chalfont, Buckinghamshire, UK). The Au surface was exposed to the 2 μL/min flow of a solution containing the dendrimer-conjugated ds-aptamers for 2 h, creating a tethered layer of the dendrimer-conjugated ds-aptamer through the thiol groups. The creation was monitored with SPR optical signal. After the tethered layer was formed, the layer was taken out of the equipment and heated to 82 °C to denature the ds-aptamers. Then, the Au surface with only the remaining single-stranded-aptamers (ss-aptamers) was transferred again to the equipment. The materials released through the denaturation were treated through the dialysis described above, in order to collect the dendrimer-conjugated single-stranded-complementary (ss-complementary).

**Figure 1:**
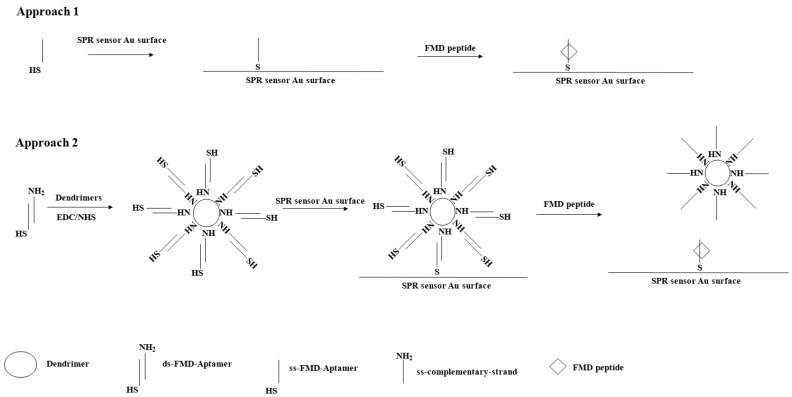
Schematic diagram for the experimental procedures.

Two different procedures were performed to collect further measurements. One was to inject only 100 μL of a FMD peptide solution into the equipment (Approach 1); the other was to inject the dendrimer-conjugated ss-complementary and the FMD peptide solution in sequence (Approach 2). The experimental procedures from the conjugation to the injections were shown in Figure 1 (next page). The injections were performed after the SPR optical signal reached a plateau, which was maintained until the release curve became constant. The SPR equipment detector was calibrated with a solution composed of 70% (*w*/*w*) glycerol.

## 3. Results

**Figure 2:**
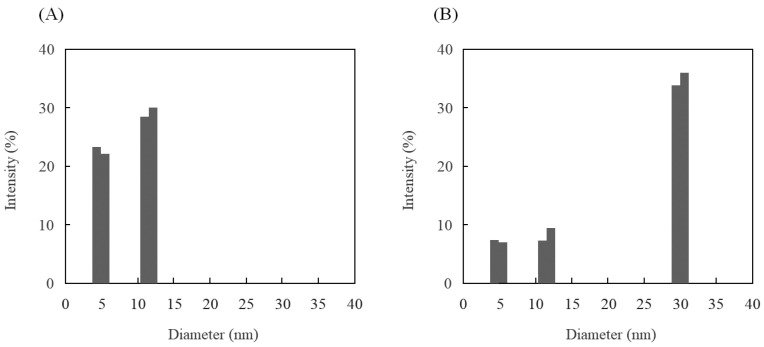
Diameter distribution before (**A**) and after (**B**) conjugation between the ds aptamers and dendrimers.

Before the conjugation, the diameters of the dendrimers and the ds aptamers were confirmed to be monodispersed at 5.0 nm and 12 nm, respectively. After the conjugation steps, the diameter distribution was found to be around 5.0, 12, and 30 nm (Figure 2). These data indicated that conjugation occurred, and the unreacted materials also remained. Therefore, the dialysis with a 1 kDa MWCO membrane was required to collect the dendrimer-conjugated ds aptamers only. After the dialysis, the diameter of the collection (30 nm) was measured for the confirmation of the conjugation. Considering the molecular weight of the dendrimer, its diameter was expected because the pure PAMAM dendrimer (molecular weight 14,215) was previously characterized as 4.5 nm [31].

**Figure 3:**
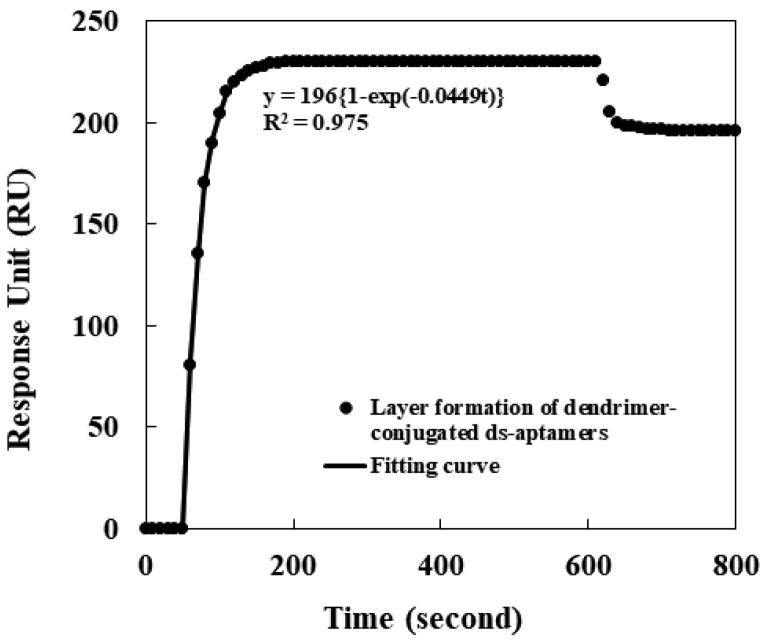
Optical signal for the layer formation of the dendrimer-conjugated ds aptamers on the SPR sensor Au surface: formation (solid circle) and fitting to the signal (solid line).

The conjugation of the dendrimers to the ds aptamers led to the summation of both molecules’ sizes (around 30 nm). The tethered layer of the dendrimer-conjugated ds aptamers was formed on the SPR sensor Au surface via a thiol bond between the aptamers and Au. The optical signal for the layer formation was observed in real-time and is shown in Figure 3 (next page). The solid circles indicate the signal for the formation, and the solid line shows fitting to the signal. The change in the signal was saturated into about 200 response units (RUs) around 180 s after the injection. The 200 RUs, corresponding to 200 ng/mm^2^, were identical to the results published previously [32,33,34]. Furthermore, the rate of the tethering was found to be proportional to the first order of space available for tethering on the Au surface, and the fitting provided the rate constant of the layer formation as 0.0449 s^−1^ with a regression coefficient of 0.975.

**Figure 4:**
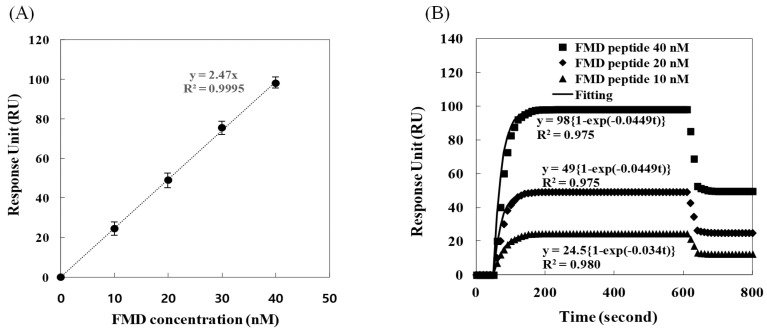
Optical signals for the correlation with FMD peptide concentration (**A**) and for the binding between FMD peptides and the ss aptamers remaining on the SPR sensor Au surface: 10, 20, and 40 nM FMD peptide concentration (triangle, diamond, and square, respectively) and fittings to the signals (solid lines) (**B**).

After the Au surface, bound with only the remaining ss-aptamers, was transferred to the SPR equipment, either only 100 μL of FMD peptides or the dendrimer-conjugated ss-complementary and FMD peptides was injected to the Au surface. For the injection of the peptide only, three different concentrations, 10, 20 and 40 nM, were considered from a previous study [11]. Since the plateau of the optical signal corresponded to the equilibrium, the difference between the plateaus represented the amount of the peptides bound to the aptamers. The comparison of the differences indicated that the change in the amplitude of the optical signal was linearly proportional to the concentration (Figure 4A). The responses were about 12, 25, and 50 RU for each concentration (triangle, diamond, and square in Figure 4B). The solid lines were the fittings to each data point. For the binding rate, the observed results were inconsistent with the prediction, because the binding rate was theoretically identical for all of concentration. The binding rate of 10 nM was 0.034 lower than the identical value, 0.0049 s^−1^, of the others. This discrepancy appears to be from the mass-transfer effect; that is, the supply of the FMD peptides to the aptamers would be more limited when the concentration was lower.

**Figure 5:**
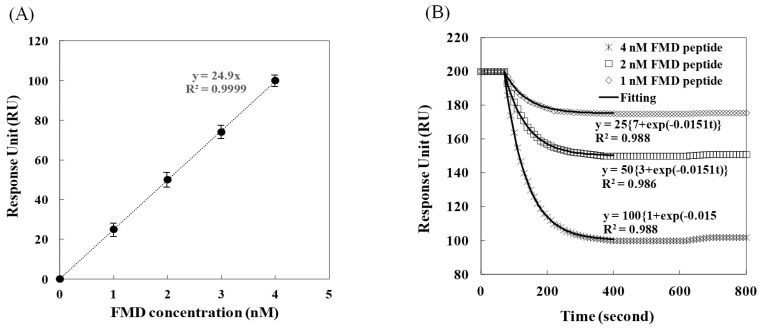
Optical signals for the correlation with the FMD peptide concentration (**A**) and for the binding between the FMD peptides and ds aptamers, which released the dendrimer-conjugated ss-complementary: 1, 2, and 4 nM FMD peptide concentrations (diamond, square, and asterisk, respectively) and fittings to the signals (solid lines) (**B**).

The dendrimer-conjugated ss-complementary collected from the dialysis was injected onto the Au surface, followed by the incubation for 2 h. The optical signal plateaued at about 200 RUs. After the incubation, the FMD peptide was injected onto the Au surface on which the dendrimer-conjugated ss-complementary was already bound to the apatamers. The concentrations of the FMD peptides were 1, 2, and 4 nM. The FMD peptides formed the specific binding with the aptamers, which led to the dissociation of the dendrimer-conjugated ss-complementary. The amplitude of the change in the optical signal decreased with the injection of the FMD peptides (Figure 5).

The amplitude of the change was also linearly proportional to the concentration (Figure 5A). The responses were about 25, 50, and 100 RUs for each concentration (diamond, square, and asterisk, respectively, in Figure 5B), which suggested that the change in the optical signal occurred at a 20-fold lower concentration compared with the FMD peptide only. Specifically, the 25 and 50 RU change, induced at 20 and 40 nM, in the injection of the FMD peptide only, were observed at 1 and 2 nM in the injections of the dendrimer-conjugated ss-complementary and the FMD peptides, respectively.

The rate constants of the binding were also different for both injections. In the injections of the dendrimer-conjugated ss-complementary-strands and the FMD peptides, the constants were 2-fold lower those in the other injection. Interestingly, the rate constants of the binding were identical for all of FMD-peptide concentrations in the injections of the dendrimer-conjugated ss-complementary-strands and the FMD peptides, whereas the constants were different in the injection of the FMD peptide only. Therefore, the low values of the rate constants in the injections of the dendrimer-conjugated ss-complementary-strands and the FMD peptides appear to be caused by the other mass-transfer effect, which might be due to the limitation caused by the steric hindrance of the conjugated dendrimers. The more amplification of the signal could be made with the bigger dendrimer. However, the slower rate seems to be inevitable due to the limitation of the movement caused by the increase in the size. However, if the decrease in the rate is desired to be minimized, the increase of the binding area may be one of the approach considered.

The limit of detection (*LOD*) was determined with the relation *LOD* = 3.3 *σ*/*S* in which *σ* is the standard deviation of four replicate responses, and *S* is the slope of the curve (Figure 4A and Figure 5A) [35]. Hence, the *LOD* obtained using the RU measurements was determined to be 0.1 and 0.01 nM for the injection of the FMD peptide only and the injections of the dendrimer-conjugated ss-complementary strands and the FMD peptides. The linear ranges were 0 to 40 and 0 to 4 nM, respectively. Recently, the FMD gene detection was investigated as an *LOD* of 0.3 fM and 0 to 1 nM of linear range using amperometry based on gold nanostructures [36,37]. However, since the detection of the genes required amplification steps that were time-consuming and equipment-demanding, the peptide detection was considered more convenient in terms of field-friendly methodology. These results indicate that the conjugation of the dendrimers is sufficiently appropriate for the detection of FMD.

## 4. Conclusions

In this study, the sensitivity in the detection of FMD peptide was improved using PAMAM dendrimers. The dendrimers were conjugated with the complementary annealed with the aptamers capable of binding specifically to FMD peptides. A tethered layer of the dendrimer-conjugated ds-aptamers was formed on the SPR sensor Au surface via a thiol bond between the aptamers and Au. After the tethered layer was formed, the Au surface was taken out of the equipment. Then, the ds-aptamers on the surface were denatured to collect the dendrimer-conjugated ss-complementary. The Au surface with only the remaining ss-aptamers was transferred again to the equipment. Two types of injection, the FMD peptide only and the dendrimer-conjugated ss-complementary followed by the FMD peptides, were performed on the Au surface. The sensitivity was increased 20 times with the conjugation of the dendrimers, but the binding rate of the peptides became more than 2 times slower due to the mass-transfer limit caused by the dendrimer hindrance. This sensitivity improvement may enable further development to detect FMD even earlier.

## Data Availability

Data are contained within the article.
